# Comparison and Reliability of Hippocampal Subfield Segmentations Within FreeSurfer Utilizing T1- and T2-Weighted Multispectral MRI Data

**DOI:** 10.3389/fnins.2021.666000

**Published:** 2021-09-08

**Authors:** René Seiger, Fabian P. Hammerle, Godber M. Godbersen, Murray B. Reed, Benjamin Spurny-Dworak, Patricia Handschuh, Manfred Klöbl, Jakob Unterholzner, Gregor Gryglewski, Thomas Vanicek, Rupert Lanzenberger

**Affiliations:** Department of Psychiatry and Psychotherapy, Medical University of Vienna, Vienna, Austria

**Keywords:** hippocampus, subfields, FreeSurfer, MRI, reliability

## Abstract

The accurate segmentation of *in vivo* magnetic resonance imaging (MRI) data is a crucial prerequisite for the reliable assessment of disease progression, patient stratification or the establishment of putative imaging biomarkers. This is especially important for the hippocampal formation, a brain area involved in memory formation and often affected by neurodegenerative or psychiatric diseases. FreeSurfer, a widely used automated segmentation software, offers hippocampal subfield delineation with multiple input options. While a single T1-weighted (T1) sequence is regularly used by most studies, it is also possible and advised to use a high-resolution T2-weighted (T2H) sequence or multispectral information. In this investigation it was determined whether there are differences in volume estimations depending on the input images and which combination of these deliver the most reliable results in each hippocampal subfield. 41 healthy participants (age = 25.2 years ± 4.2 SD) underwent two structural MRIs at three Tesla (time between scans: 23 days ± 11 SD) using three different structural MRI sequences, to test five different input configurations (T1, T2, T2H, T1 and T2, and T1 and T2H). We compared the different processing pipelines in a cross-sectional manner and assessed reliability using test-retest variability (%TRV) and the dice coefficient. Our analyses showed pronounced significant differences and large effect sizes between the processing pipelines in several subfields, such as the molecular layer (head), CA1 (head), hippocampal fissure, CA3 (head and body), fimbria and CA4 (head). The longitudinal analysis revealed that T1 and multispectral analysis (T1 and T2H) showed overall higher reliability across all subfields than T2H alone. However, the specific subfields had a substantial influence on the performance of segmentation results, regardless of the processing pipeline. Although T1 showed good test-retest metrics, results must be interpreted with caution, as a standard T1 sequence relies heavily on prior information of the atlas and does not take the actual fine structures of the hippocampus into account. For the most accurate segmentation, we advise the use of multispectral information by using a combination of T1 and high-resolution T2-weighted sequences or a T2 high-resolution sequence alone.

## Introduction

Following the seminal findings from Scoville and Milner studying the “patient H. M.” (Scoville and Milner, 1957) the hippocampus has become one of the most investigated brain regions related to memory processing (Bird and Burgess, 2008), learning (Brasted et al., 2003), or spatial navigation (O’Keefe and Nadel, 1978; O’Keefe et al., 1998). It is one of the few brain regions where adult neurogenesis occurs (Eriksson et al., 1998; Toda et al., 2019) and is highly susceptible to actions related to neuroplasticity (MacQueen and Frodl, 2011; Kraus et al., 2017). On the contrary, for example, it is one of the first brain structures affected in dementia by the accumulation of neurofibrillary tangles (Braak and Braak, 1991) and highly vulnerable to chronic stress such as in psychiatric conditions (Geuze et al., 2005), as repeatedly demonstrated in major depressive disorder (Frodl et al., 2002; Campbell et al., 2004; Videbech and Ravnkilde, 2004; Arnone et al., 2013; Schmaal et al., 2016). Interestingly, therapeutic intervention seems to restore gray matter configurations back to regular levels (Sartorius et al., 2016).

The hippocampus is not a homogenous brain structure, as it consists of distinct subfields, with specific cell properties which are functionally segregated (Duvernoy et al., 2013) as reflected in the trisynaptic circuit (Samuels et al., 2015). Input from the entorhinal cortex enters granule cells in dentate gyrus over the perforant pathway. Mossy fibers from the dentate gyrus project to CA3 pyramidal cells, while CA3 neurons send their information to pyramidal cells of CA1 *via* the Schaffer collaterals, where information is sent back to the subiculum and the entorhinal cortex (Andersen et al., 2006; Stepan et al., 2015). It has been shown that the dentate gyrus is involved in pattern separation, the CA3 in pattern completion, CA1 in input integration and the subiculum in memory retrieval (Zeineh et al., 2003; Lee et al., 2004; Leutgeb et al., 2004; Bakker et al., 2008; Small et al., 2011). Each subfield is specifically affected by certain diseases, as outlined in Small et al. (2011). For example, while in Alzheimer’s disease (AD) the entorhinal cortex and to some extent CA1 and the subiculum are most affected, in depression predominantly the subiculum and to some extent the CA1 were most susceptible. Interestingly, the dentate gyrus seems to be largely unaffected by AD. The same is seen in temporal lobe epilepsy (TLE) with mesial temporal sclerosis (TLE-MTS) where an overall decline in the subfields is observed, but not in the subiculum, which is quite different to the pattern seen in AD or depression (West et al., 1994; Gomez-Isla et al., 1996; Posener et al., 2003; Ballmaier et al., 2008; Mueller et al., 2009; Small, 2014). These findings grant valuable information to better monitor disease progression, onset and also for the putative development of biomarkers or prognosis for treatment outcome, specifically tailored for the respective disease. Therefore, reliable assessment of these hippocampal substructures and high reliability of processing strategies are of utmost importance for human *in vivo* neuroscientific investigations.

Fast progress has been made in the development of automated hippocampal segmentation methods within different software packages enabling distinct subfield segmentations (Pipitone et al., 2014; Iglesias et al., 2015; Yushkevich et al., 2015). Currently, the FreeSurfer software^1^ with its dedicated hippocampal subfield tool is most frequently used (Iglesias et al., 2015). Several studies have already applied this approach to their research in different contexts, for recent examples see Gryglewski et al. (2019); Kraus et al. (2019); Dounavi et al. (2020); and van Eijk et al. (2020). The latest hippocampus segmentation tool, now part of the FreeSurfer 7 release, uses a probabilistic atlas built from *ex vivo* magnetic resonance imaging (MRI) data recorded at 0.13 mm isotropic resolution from 15 autopsy brains and *in vivo* information. The *in vivo* data recorded at standard resolution was used to account for neighboring structures of the hippocampus. A generative framework is used to handle MRI data with different contrast properties, hence either mono- or multispectral information can be taken as input. The final estimation of the hippocampal subfield volumes is carried out by using a Bayesian inference approach (Iglesias et al., 2015).

Usually a T1-weighted sequence is used for whole-brain image analysis techniques such as voxel-based morphometry or cortical thickness assessments (Hutton et al., 2009). However, due to the complex structure of the hippocampus and its composition of different cell compartments, high resolution T2 images have been shown to deliver better and more suitable contrast properties for hippocampal subfield delineation (Winterburn et al., 2013). This has been corroborated by a recent study where T2-high resolution scans outperformed T1 images in terms of disease status detection (Mueller et al., 2018). Furthermore, some hippocampal regions, such as the molecular layer, fimbria or the fissure show low test-retest reliability or cannot be delineated based on the contrast properties of a T1 sequence alone (Whelan et al., 2016; Brown et al., 2020) while it should deliver better results using a high-resolution T2-weighted sequence (Iglesias et al., 2015).

The hippocampus tool in FreeSurfer offers the possibility to use a single (e.g., T1) or multispectral input (e.g., T1 and T2H) for delineation of the hippocampus. Despite the putative benefits of T2H and multispectral processing, some issues must be considered. First, recording an additional structural sequence is time consuming. This is especially relevant for clinical settings where patients are measured. However, this is mandatory in FreeSurfer, as all subjects have to be processed first with the regular T1-weighted recon-all stream prior to hippocampal subfield analysis. This requires at least two sequences to be recorded, if the subfield tool is used with an additional scan, or a scan different than T1-weighted. Secondly, high resolution T2-weighted sequence parameters need technical expertise, which is not available in all laboratories. In addition, the correct application of the T2H field of view (FOV) prior the measurement is also crucial and has to be performed precisely, as the sequences FOV barely covers the hippocampal structure along the main axis, due to scanner restraints imposed by the high-resolution sequence.

To investigate if the effort or drawbacks justify the gained improved signal quality, we conducted a systematic comparison of the different processing modes available within FreeSurfer. All participants were measured twice in a longitudinal setting with three different MR sequences at each time point (TP) (T1, T2 and high-resolution T2). We assessed five possible input configurations available within the recently released FreeSurfer 7: Whole-brain T1-weighted (T1), whole-brain T2-weighted (T2), T2-weighted high-resolution for hippocampus only (T2H) and combination *via* multispectral analysis of T1 and T2 and T1 and T2H were compared cross-sectionally. Within the same population of subjects, we assessed if these different combinations of sequences deliver different volume estimations for each subfield. Subsequently, test-retest analyses were performed within the same subjects for all subfields within FreeSurfer to assess which sequence or sequence combination delivers the most reliable values.

## Materials and Methods

### Subjects and Study Design

41 right-handed healthy participants (age = 25.2 years ± 4.2 SD, 30 females) were included in this investigation. All subjects underwent two structural MRI measurements approximately 3 weeks apart (23 days ± 11 SD). Screening for general health was carried out prior to study inclusion and comprised medical history assessment, a physical examination and the structured clinical interview for DSM-IV (SCID) to rule out physical and mental disorders. Exclusion criteria comprised any medical, psychiatric or neurological illness, current or former substance abuse, MRI contraindications, pregnancy, first degree relatives with a history of psychiatric illness and smoking. Recruitment was conducted through flyers at the Department of Psychiatry and Psychotherapy at the Medical University of Vienna. This study was approved by the ethical committee of the Medical University of Vienna and was performed in accordance with the Declaration of Helsinki (1964). All participants gave written informed consent to participate in this study. Data is taken from a study registered at clinicaltrials.gov with the identifier NCT02753738.

### Data Acquisition

Structural MRI scans were recorded with a 3T Siemens Prisma scanner using a 64-channel head coil and the following three sequences: a whole-brain T1-weighted scan [Repetition time (TR) = 2,300 ms; echo time (TE) = 2.95 ms; inversion time (TI) = 900 ms; flip angle (α) = 9°; matrix = 240 × 256, 176 slices; 1.05 × 1.05 × 1.20 mm^3^; acquisition time (TA) = 5:09 min], a whole-brain T2-weighted scan (TR = 3,200 ms; TE = 408 ms; α = 120°; matrix = 256 × 256, 192 slices; 0.9 × 0.9 × 0.9 mm^3^; TA = 5:01 min) and a high resolution T2-weighted scan covering both hippocampi (TR = 8,000 ms; TE = 51 ms; matrix = 448 × 448, 40 slices; α = 133°; 0.39 × 0.39 × 1.20 mm^3^; TA = 7:52).

### Data Processing

After a visual quality check of the MRI data, subjects were initially processed with the FreeSurfer 6.0 (see text footnote 1) “recon-all” standard pipeline (Dale et al., 1999; Fischl et al., 1999) for the cross-sectional comparison. In general, Talairach registration (Talairach and Tournoux, 1988), correction for bias field and skull stripping (Ségonne et al., 2004) is performed. This is followed by segmentation of white and gray matter areas (Fischl et al., 2002, 2004) and calculation of white and pial surfaces (Fischl and Dale, 2000).

In addition, all subjects were processed with the longitudinal recon-all stream (Reuter et al., 2012) for the assessment of the test-retest metrics. Here, a subject specific template is created of the two TP using robust, inverse consistent registration (Reuter et al., 2010). Information from this within-subject template is then utilized for the initialization of further processing steps (Reuter et al., 2012). For applications and a detailed description of both “recon-all” processing pipelines please see prior publications by our group (Seiger et al., 2016, 2018).

**FIGURE 1:**
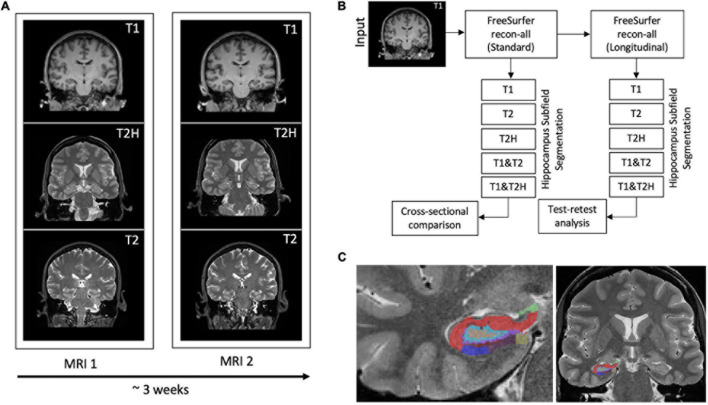
**(A)** Three different MRI sequences (T1: T1-weighted, T2H: T2-weighted high-resolution and T2: T2-weighted standard resolution) were recorded for each subject at baseline and after approximately 3 weeks. **(B)** Depiction of the processing scheme. The T1-weighted sequence is used for the standard pipeline within FreeSurfer. All data was subsequently processed with the longitudinal stream. Hippocampus segmentation was then performed with the five different input configurations using the cross-sectionally as well as the longitudinally processed data to conduct the cross-sectional comparison and the test-retest analysis. **(C)** Representative hippocampal segmentation of a study participant using the high-resolution T2-weighted sequence.

This was followed by the new hippocampal subfield segmentation approach. In this investigation, the hippocampal tool from the development version (20191217) was used, which is now available in FreeSurfer 7 (Iglesias et al., 2015). This tool segments the different subfields by using a Bayesian inference approach based on image intensities and prior knowledge of a probabilistic atlas which was generated of *in vivo* manual segmentations and ultra-high resolution *ex vivo* MRI data (Van Leemput, 2009; Iglesias et al., 2016). Subsequently, subfield volumes were calculated using five different input configurations. First, the standard T1 image was used, followed by a solely usage of the high-resolution T2 (T2H) and the T2 only scan. In addition, multispectral analysis was performed by calculating the subfields using information by combining T1 and T2H and T1 and T2. Finally, 22 regions of interest (ROIs) (19 subfields with head and body subdivisions and the whole hippocampus with head and body subdivisions) per hemisphere were extracted. After processing, data of all subjects were visually inspected to check for putative misclassifications or processing errors in general. After our inspection, no processing errors were detected and all data could be used for subsequent analyses. Detailed processing steps are depicted in Figure 1.

### Statistical Analysis

Statistical analyses were carried out with the R software (R Core Team, 2019) and MATLAB R2014a (The MathWorks, Natick, MA, United States). To assess significant differences and effect sizes between the five processing types (T1, T2, T2H, T1 and T2, and T1 and T2H), cross-sectionally processed subfields were analyzed using non-parametric Friedman tests with Kendall’s W. Pairwise Wilcoxon signed-rank tests with Bonferroni correction were further used for *post hoc* analyses. All these tests were performed for each of the 22 ROIs. For test-retest performance, percentage test-retest variability (%TRV):

$$\%TRV=\frac{\left|T\,P\,1-T\,P\,2\right|}{\left(\frac{T\,P\,1+T\,P\,2}{2}\right)}*100$$

and Dice coefficients:

$$\mathrm{Dice}\,\mathrm{coefficent}=\frac{2*\left|T\,P\,1\cap T\,P\,2\right|}{\left|T\,P\,1\right|+\left|T\,P\,2\right|}$$

were calculated using the longitudinally processed “recon-all” data of the two TP within FreeSurfer.

## Results

**FIGURE 2:**
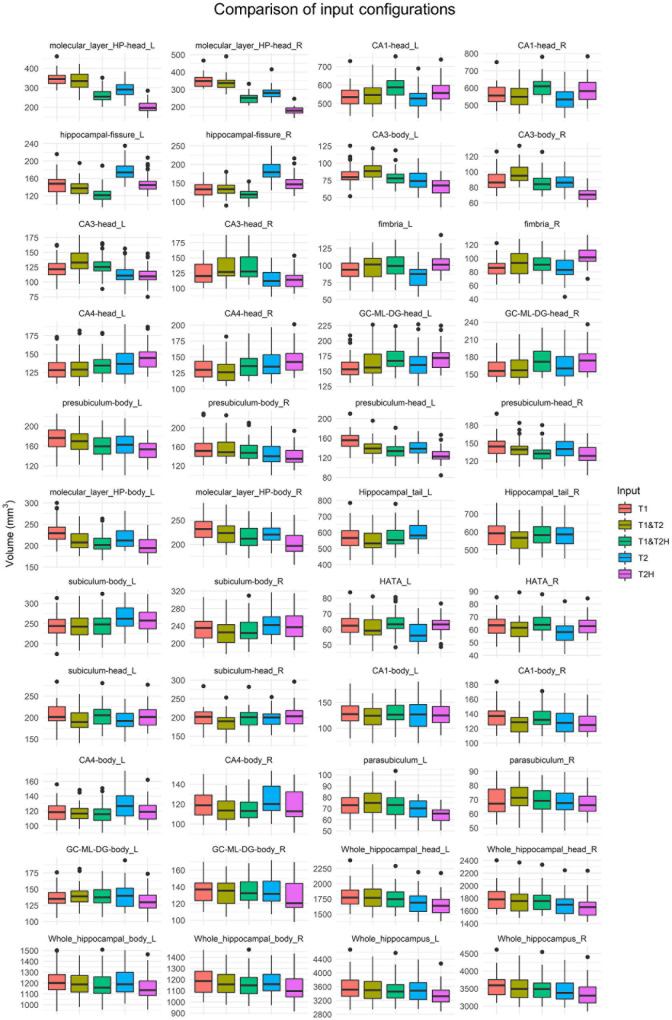
Boxplots showing volume estimations of the cross-sectional hippocampal subfield investigation using five different input configurations (T1, T1 and T2, T1 and T2H, T2, and T2H). Subfields are arranged according to the height of effect sizes of the Friedman test (X^2^) using Kendall’s W. In addition to the 19 ROIs, whole hippocampal head and body as well as whole hippocampal volume are presented. All subfields showed significant differences according to the Friedman tests (see Table 1). T2H was excluded for the hippocampal tail, as not the entire structure was covered due to the limited size of the field of view. T2, T2-weighted standard resolution; T2H, T2-weighted high resolution; GC-ML-DG, granule cell and molecular layer of the dentate gyrus; HATA, hippocampus-amygdala-transition-area.

The Friedman tests with Wilcoxon *post hoc* tests conducted for the cross-sectional analysis comprising the five different processing pipelines (T1, T2, T2H, T1 and T2, and T1 and T2H) revealed vast significant differences in several subfields between the input configurations. The greatest volume differences between processing types in terms of effect sizes were observed in the head of the molecular layer, head of CA1, hippocampal fissure, head and body of CA3, fimbria and head of CA4 (for detailed results of Friedman tests, *post hoc* analyses and boxplots see Figure 2 and Table 1). Further analysis of these regions showed subfield specific differences regarding the mode of processing. For example, while T2H led to lowest volume estimations in the head of the molecular layer (192.11 ± 24.74; mean ± SD, T1: 349.80 ± 34.61), T2 showed highest values in the hippocampal fissure (180.88 ± 21.98, T1: 140.26 ± 19.97) and was significantly different to the other processing modes within this subfield. However, as for the molecular layer, all processing modes strongly differed from each other in this subfield. The CA3 body also showed lowest values for T2H (68.89 ± 9.87, T1: 86.45 ± 12.22), similar to the head of the molecular layer. Furthermore, significant differences for the whole hippocampus were observed, with highest values for T1 in comparison to the other processing types (3,588.62 ± 336.41) (see Figure 2).

**TABLE 1:** Friedman tests with pairwise Wilcoxon *post hoc* comparisons. Subfields are arranged according to the height of effect sizes of the Friedman test (X^2^) using Kendall’s W.

Friedman Test		Post-hoc Wilcoxon signed-rank test								
		Pairwise comparison			Left Hippocampus			Right Hippocampus		
					Statistics	P-adjusted	sig.	Statistics	P-adjusted	sig.
**molecular_layer_HP-head**		T1	–	T1&T2	637	1	n.s.	661	0.864	n.s.
X^2^_(Left)_ =	149.46	T1	–	T1&T2H	861	3.927E-10	***	861	3.927E-10	***
pvalue_(Left)_ =	2.65E-31	T1	–	T2	859	1.179E-09	***	861	3.927E-10	***
W_(Left)_ =	0.91	T1	–	T2H	861	3.927E-10	***	861	3.927E-10	***
		T1&T2	–	T1&T2H	860	7.862E-10	***	861	3.927E-10	***
X^2^_(Right)_ =	155.38	T1&T2	–	T2	830	9.331E-07	***	861	3.927E-10	***
pvalue_(Right)_ =	1.43E-32	T1&T2	–	T2H	861	3.927E-10	***	861	3.927E-10	***
W_(Right)_ =	0.95	T1&T2H	–	T2	33	1.261E-06	***	31	9.331E-07	***
		T1&T2H	–	T2H	861	3.927E-10	***	861	3.927E-10	***
		T2	–	T2H	861	3.927E-10	***	861	3.927E-10	***

**CA1-head**		T1	–	T1&T2	261	1	n.s.	719.5	0.07992	n.s.
X^2^_(Left)_ =	124.35	T1	–	T1&T2H	0	3.927E-10	***	0	3.927E-10	***
pvalue_(Left)_ =	6.28E-26	T1	–	T2	729	0.0199152	n.s.	847	4.32E-08	***
W_(Left)_ =	0.76	T1	–	T2H	34	1.464E-06	***	36	1.957E-06	***
		T1&T2	–	T1&T2H	3	1.966E-09	***	0	3.927E-10	***
X^2^_(Right)_ =	138.60	T1&T2	–	T2	828	1.261E-06	***	783	0.0002532	***
pvalue_(Right)_ =	5.64E-29	T1&T2	–	T2H	114	0.0054432	n.s.	1	7.862E-10	***
W_(Right)_ =	0.85	T1&T2H	–	T2	861	3.927E-10	***	861	3.927E-10	***
		T1&T2H	–	T2H	821	3.417E-06	***	785	0.0002087	***
		T2	–	T2H	1	7.862E-10	***	0	3.927E-10	***

**hippocampal-fissure**		T1	–	T1&T2	784	0.0002298	***	450	1	n.s.
X^2^_(Left)_ =	128.31	T1	–	T1&T2H	860	7.862E-10	***	846	5.4E-08	***
pvalue_(Left)_ =	8.94E-27	T1	–	T2	16	6.653E-08	***	0	3.927E-10	***
W_(Left)_ =	0.78	T1	–	T2H	372	1	n.s.	85	0.0004882	***
		T1&T2	–	T1&T2H	847	4.32E-08	***	832	6.826E-07	***
X^2^_(Right)_ =	128.06	T1&T2	–	T2	0	3.927E-10	***	0	3.927E-10	***
pvalue_(Right)_ =	1.01E-26	T1&T2	–	T2H	141	0.0362448	n.s.	101	0.0019526	n.s.
W_(Right)_ =	0.78	T1&T2H	–	T2	0	3.927E-10	***	0	3.927E-10	***
		T1&T2H	–	T2H	13	3.456E-08	***	0	3.927E-10	***
		T2	–	T2H	834	4.968E-07	***	846	5.4E-08	***

**CA3-body**		T1	–	T1&T2	42	4.493E-06	***	95	0.0011794	n.s.
X^2^_(Left)_ =	123.82	T1	–	T1&T2H	701	0.117504	n.s.	735	0.0130896	n.s.
pvalue_(Left)_ =	8.14E-26	T1	–	T2	718	0.041256	n.s.	584	1	n.s.
W_(Left)_ =	0.76	T1	–	T2H	860	7.862E-10	***	861	3.927E-10	***
		T1&T2	–	T1&T2H	861	3.927E-10	***	842	1.205E-07	***
X^2^_(Right)_ =	118.40	T1&T2	–	T2	838	2.514E-07	***	821	3.417E-06	***
pvalue_(Right)_ =	1.17E-24	T1&T2	–	T2H	861	3.927E-10	***	861	3.927E-10	***
W_(Right)_ =	0.72	T1&T2H	–	T2	571	1	n.s.	388	1	n.s.
		T1&T2H	–	T2H	858	1.966E-09	***	861	3.927E-10	***
		T2	–	T2H	857	2.752E-09	***	861	3.927E-10	***

**CA3-head**		T1	–	T1&T2	12	2.752E-08	***	114	0.0054432	n.s.
X^2^_(Left)_ =	120.55	T1	–	T1&T2H	110	0.0040046	n.s.	37	2.255E-06	***
pvalue_(Left)_ =	4.08E-25	T1	–	T2	766	0.0011794	n.s.	796	6.826E-05	***
W_(Left)_ =	0.74	T1	–	T2H	815	7.517E-06	***	804	2.825E-05	***
		T1&T2	–	T1&T2H	789	0.0001404	***	480	1	n.s.
X^2^_(Right)_ =	111.98	T1&T2	–	T2	861	3.927E-10	***	861	3.927E-10	***
pvalue_(Right)_ =	2.75E-23	T1&T2	–	T2H	861	3.927E-10	***	858	1.966E-09	***
W_(Right)_ =	0.68	T1&T2H	–	T2	851	1.689E-08	***	851	1.689E-08	***
		T1&T2H	–	T2H	861	3.927E-10	***	861	3.927E-10	***
		T2	–	T2H	683	0.32184	n.s.	399	1	n.s.

**fimbria**		T1	–	T1&T2	151	0.068256	n.s.	72	0.0001404	***
X^2^_(Left)_ =	88.88	T1	–	T1&T2H	36	1.957E-06	***	65	6.826E-05	***
pvalue_(Left)_ =	2.28E-18	T1	–	T2	749	0.0046656	n.s.	518	1	n.s.
W_(Left)_ =	0.54	T1	–	T2H	45	6.61E-06	***	2	1.179E-09	***
		T1&T2	–	T1&T2H	339	1	n.s.	591	1	n.s.
X^2^_(Right)_ =	96.02	T1&T2	–	T2	843	9.936E-08	***	802	3.542E-05	***
pvalue_(Right)_ =	6.92E-20	T1&T2	–	T2H	259	1	n.s.	62	4.925E-05	***
W_(Right)_ =	0.59	T1&T2H	–	T2	811	1.236E-05	***	696	0.156816	n.s.
		T1&T2H	–	T2H	318	1	n.s.	22	2.104E-07	***
		T2	–	T2H	3	1.966E-09	***	0	3.927E-10	***

**CA4-head**		T1	–	T1&T2	380	1	n.s.	633	1	n.s.
X^2^_(Left)_ =	89.11	T1	–	T1&T2H	101	0.0019526	n.s.	59	3.542E-05	***
pvalue_(Left)_ =	2.03E-18	T1	–	T2	58	3.162E-05	***	107	0.0031622	n.s.
W_(Left)_ =	0.54	T1	–	T2H	0	3.927E-10	***	16	6.653E-08	***
		T1&T2	–	T1&T2H	161	0.124416	n.s.	16	6.653E-08	***
X^2^_(Right)_ =	83.08	T1&T2	–	T2	34	1.464E-06	***	20	1.456E-07	***
pvalue_(Right)_ =	3.87E-17	T1&T2	–	T2H	22	2.104E-07	***	4	2.752E-09	***
W_(Right)_ =	0.51	T1&T2H	–	T2	232	1	n.s.	304	1	n.s.
		T1&T2H	–	T2H	25	3.551E-07	***	86	0.0005357	***
		T2	–	T2H	169	0.19656	n.s.	170	0.208224	n.s.

**GC-ML-DG-head**		T1	–	T1&T2	217	1	n.s.	391	1	n.s.
X^2^_(Left)_ =	71.10	T1	–	T1&T2H	2	1.179E-09	***	0	3.927E-10	***
pvalue_(Left)_ =	1.33E-14	T1	–	T2	142	0.0387072	n.s.	196	0.864	n.s.
W_(Left)_ =	0.43	T1	–	T2H	0	3.927E-10	***	12	2.752E-08	***
		T1&T2	–	T1&T2H	88	0.0006394	***	6	5.486E-09	***
X^2^_(Right)_ =	93.48	T1&T2	–	T2	376	1	n.s.	156	0.092448	n.s.
pvalue_(Right)_ =	2.40E-19	T1&T2	–	T2H	113	0.0050544	n.s.	31	9.331E-07	***
W_(Right)_ =	0.57	T1&T2H	–	T2	711	0.064368	n.s.	792	0.0001037	***
		T1&T2H	–	T2H	366	1	n.s.	568	1	n.s.
		T2	–	T2H	89	0.0006998	***	98	0.0015206	n.s.

**presubiculum-body**		T1	–	T1&T2	552	1	n.s.	463	1	n.s.
X^2^_(Left)_ =	79.22	T1	–	T1&T2H	845	6.653E-08	***	776	0.0004882	***
pvalue_(Left)_ =	2.55E-16	T1	–	T2	689	0.232416	n.s.	746	0.0058752	n.s.
W_(Left)_ =	0.48	T1	–	T2H	860	7.862E-10	***	860	7.862E-10	***
		T1&T2	–	T1&T2H	802	3.542E-05	***	729	0.0199152	n.s.
X^2^_(Right)_ =	82.22	T1&T2	–	T2	685	0.289008	n.s.	758	0.0022982	n.s.
pvalue_(Right)_ =	5.88E-17	T1&T2	–	T2H	830	9.331E-07	***	842	1.205E-07	***
W_(Right)_ =	0.50	T1&T2H	–	T2	275	1	n.s.	609	1	n.s.
		T1&T2H	–	T2H	803	3.162E-05	***	855	5.486E-09	***
		T2	–	T2H	786	0.0001892	***	597	1	n.s.

**presubiculum-head**		T1	–	T1&T2	840	1.758E-07	***	728	0.0212976	n.s.
X^2^_(Left)_ =	96.45	T1	–	T1&T2H	861	3.927E-10	***	848	3.456E-08	***
pvalue_(Left)_ =	5.61E-20	T1	–	T2	827	1.464E-06	***	560	1	n.s.
W_(Left)_ =	0.59	T1	–	T2H	860	7.862E-10	***	827	1.464E-06	***
		T1&T2	–	T1&T2H	693	0.186192	n.s.	779	0.0003698	***
X^2^_(Right)_ =	62.20	T1&T2	–	T2	407	1	n.s.	332	1	n.s.
pvalue_(Right)_ =	9.98E-13	T1&T2	–	T2H	813	9.677E-06	***	726	0.0243648	n.s.
W_(Right)_ =	0.38	T1&T2H	–	T2	196	0.864	n.s.	148	0.056592	n.s.
		T1&T2H	–	T2H	780	0.000337	***	505	1	n.s.
		T2	–	T2H	824	2.255E-06	***	776	0.0004882	***

**molecular_layer_HP-body**		T1	–	T1&T2	831	7.992E-07	***	684	0.304992	n.s.
X^2^_(Left)_ =	83.18	T1	–	T1&T2H	852	1.296E-08	***	844	8.122E-08	***
pvalue_(Left)_ =	3.69E-17	T1	–	T2	790	0.000127	***	737	0.0113616	n.s.
W_(Left)_ =	0.51	T1	–	T2H	836	3.551E-07	***	858	1.966E-09	***
		T1&T2	–	T1&T2H	627	1	n.s.	688	0.245808	n.s.
X^2^_(Right)_ =	74.60	T1&T2	–	T2	252	1	n.s.	482	1	n.s.
pvalue_(Right)_ =	2.43E-15	T1&T2	–	T2H	684	0.304992	n.s.	789	0.0001404	***
W_(Right)_ =	0.45	T1&T2H	–	T2	118	0.007344	n.s.	197	0.864	n.s.
		T1&T2H	–	T2H	675	0.432	n.s.	770	0.0008338	***
		T2	–	T2H	780	0.000337	***	828	1.261E-06	***

**Hippocampal_tail**		T1	–	T1&T2	773	0.0006394	***	837	2.994E-07	***
X^2^_(Left)_ =	58.11	T1	–	T1&T2H	393	1	n.s.	670	0.432	n.s.
pvalue_(Left)_ =	1.49E-12	T1	–	T2	160	0.117504	n.s.	551	1	n.s.
W_(Left)_ =	0.47	T1&T2	–	T1&T2H	35	1.693E-06	***	47	8.554E-06	***
		T1&T2	–	T2	4	2.752E-09	***	96	0.001283	n.s.
X^2^_(Right)_ =	51.94	T1&T2H	–	T2	142	0.0387072	n.s.	460	1	n.s.
pvalue_(Right)_ =	3.09E-11									
W_(Right)_ =	0.42									

**subiculum-body**		T1	–	T1&T2	531	1	n.s.	768	0.0009936	***
X^2^_(Left)_ =	79.86	T1	–	T1&T2H	356	1	n.s.	613	1	n.s.
pvalue_(Left)_ =	1.86E-16	T1	–	T2	22	2.104E-07	***	199	0.864	n.s.
W_(Left)_ =	0.49	T1	–	T2H	82	0.0003698	***	341	1	n.s.
		T1&T2	–	T1&T2H	242	1	n.s.	206	1	n.s.
X^2^_(Right)_ =	55.00	T1&T2	–	T2	13	3.456E-08	***	20	1.456E-07	***
pvalue_(Right)_ =	3.24E-11	T1&T2	–	T2H	56	2.519E-05	***	106	0.0029246	n.s.
W_(Right)_ =	0.34	T1&T2H	–	T2	38	2.592E-06	***	13	3.456E-08	***
		T1&T2H	–	T2H	34	1.464E-06	***	92	0.0009115	***
		T2	–	T2H	574	1	n.s.	602	1	n.s.

**HATA**		T1	–	T1&T2	651	1	n.s.	668	0.864	n.s.
X^2^_(Left)_ =	46.01	T1	–	T1&T2H	225	1	n.s.	187	0.432	n.s.
pvalue_(Left)_ =	2.45E-09	T1	–	T2	779	0.0003698	***	806	2.242E-05	***
W_(Left)_ =	0.28	T1	–	T2H	466	1	n.s.	412	1	n.s.
		T1&T2	–	T1&T2H	119	0.0079056	n.s.	67	8.424E-05	***
X^2^_(Right)_ =	62.67	T1&T2	–	T2	754	0.0031622	n.s.	679	0.397872	n.s.
pvalue_(Right)_ =	7.95E-13	T1&T2	–	T2H	308	1	n.s.	190	0.432	n.s.
W_(Right)_ =	0.38	T1&T2H	–	T2	810	1.395E-05	***	838	2.514E-07	***
		T1&T2H	–	T2H	640	1	n.s.	622	1	n.s.
		T2	–	T2H	102	0.0021168	n.s.	56	2.519E-05	***

**subiculum-head**		T1	–	T1&T2	816	6.61E-06	***	726	0.0243648	n.s.
X^2^_(Left)_ =	54.19	T1	–	T1&T2H	524	1	n.s.	461	1	n.s.
pvalue_(Left)_ =	4.81E-11	T1	–	T2	773	0.0006394	***	475	1	n.s.
W_(Left)_ =	0.33	T1	–	T2H	516	1	n.s.	241	1	n.s.
		T1&T2	–	T1&T2H	59	3.542E-05	***	78	0.0002532	***
X^2^_(Right)_ =	44.10	T1&T2	–	T2	398	1	n.s.	158	0.104112	n.s.
pvalue_(Right)_ =	6.12E-09	T1&T2	–	T2H	122	0.0098496	n.s.	83	0.0004061	***
W_(Right)_ =	0.27	T1&T2H	–	T2	740	0.0091584	n.s.	468	1	n.s.
		T1&T2H	–	T2H	470	1	n.s.	215	1	n.s.
		T2	–	T2H	136	0.0260928	n.s.	219	1	n.s.

**CA1-body**		T1	–	T1&T2	696	0.156816	n.s.	809	1.577E-05	***
X^2^_(Left)_ =	33.99	T1	–	T1&T2H	283	1	n.s.	537	1	n.s.
pvalue_(Left)_ =	7.49E-07	T1	–	T2	501	1	n.s.	727	0.0228096	n.s.
W_(Left)_ =	0.21	T1	–	T2H	440	1	n.s.	757	0.0024883	n.s.
		T1&T2	–	T1&T2H	35	1.693E-06	***	45	6.61E-06	***
X^2^_(Right)_ =	61.50	T1&T2	–	T2	138	0.029808	n.s.	223	1	n.s.
pvalue_(Right)_ =	1.40E-12	T1&T2	–	T2H	136	0.0260928	n.s.	301	1	n.s.
W_(Right)_ =	0.38	T1&T2H	–	T2	620	1	n.s.	701	0.117504	n.s.
		T1&T2H	–	T2H	610	1	n.s.	840	1.758E-07	***
		T2	–	T2H	393	1	n.s.	485	1	n.s.

**CA4-body**		T1	–	T1&T2	607	1	n.s.	750	0.00432	n.s.
X^2^_(Left)_ =	37.93	T1	–	T1&T2H	659	0.864	n.s.	713	0.056592	n.s.
pvalue_(Left)_ =	1.16E-07	T1	–	T2	143	0.041256	n.s.	232	1	n.s.
W_(Left)_ =	0.23	T1	–	T2H	399	1	n.s.	525	1	n.s.
		T1&T2	–	T1&T2H	538	1	n.s.	292	1	n.s.
X^2^_(Right)_ =	46.81	T1&T2	–	T2	23	2.514E-07	***	18	9.936E-08	***
pvalue_(Right)_ =	1.67E-09	T1&T2	–	T2H	296	1	n.s.	283	1	n.s.
W_(Right)_ =	0.29	T1&T2H	–	T2	40	3.417E-06	***	51	1.395E-05	***
		T1&T2H	–	T2H	198	0.864	n.s.	332	1	n.s.
		T2	–	T2H	795	0.0000756	***	814	8.554E-06	***

**parasubiculum**		T1	–	T1&T2	340	1	n.s.	206	1	n.s.
X^2^_(Left)_ =	42.91	T1	–	T1&T2H	512	1	n.s.	263	1	n.s.
pvalue_(Left)_ =	1.08E-08	T1	–	T2	623	1	n.s.	412	1	n.s.
W_(Left)_ =	0.26	T1	–	T2H	800	4.406E-05	***	463	1	n.s.
		T1&T2	–	T1&T2H	688	0.245808	n.s.	706	0.087264	n.s.
X^2^_(Right)_ =	21.40	T1&T2	–	T2	734	0.0140832	n.s.	644	1	n.s.
pvalue_(Right)_ =	0.0002632	T1&T2	–	T2H	797	6.134E-05	***	656	1	n.s.
W_(Right)_ =	0.13	T1&T2H	–	T2	589	1	n.s.	508	1	n.s.
		T1&T2H	–	T2H	778	0.0004061	***	572	1	n.s.
		T2	–	T2H	805	2.519E-05	***	533	1	n.s.

**GC-ML-DG-body**		T1	–	T1&T2	292	1	n.s.	532	1	n.s.
X^2^_(Left)_ =	29.44	T1	–	T1&T2H	268	1	n.s.	405	1	n.s.
pvalue_(Left)_ =	6.35E-06	T1	–	T2	314	1	n.s.	434	1	n.s.
W_(Left)_ =	0.18	T1	–	T2H	641	1	n.s.	735	0.0130896	n.s.
		T1&T2	–	T1&T2H	414	1	n.s.	326	1	n.s.
X^2^_(Right)_ =	31.67	T1&T2	–	T2	387	1	n.s.	343	1	n.s.
pvalue_(Right)_ =	2.24E-06	T1&T2	–	T2H	746	0.0058752	n.s.	700	0.124416	n.s.
W_(Right)_ =	0.19	T1&T2H	–	T2	384	1	n.s.	460	1	n.s.
		T1&T2H	–	T2H	796	6.826E-05	***	813	9.677E-06	***
		T2	–	T2H	807	1.996E-05	***	834	4.968E-07	***
**Whole_hippocampal_head**		T1	–	T1&T2	612	1	n.s.	780	0.000337	***
X^2^_(Left)_ =	117.05	T1	–	T1&T2H	817	5.832E-06	***	840	1.758E-07	***
pvalue_(Left)_ =	2.27E-24	T1	–	T2	851	1.689E-08	***	851	1.689E-08	***
W_(Left)_ =	0.71	T1	–	T2H	861	3.927E-10	***	861	3.927E-10	***
		T1&T2	–	T1&T2H	670	0.432	n.s.	457	1	n.s.
X^2^_(Right)_ =	124.18	T1&T2	–	T2	860	7.862E-10	***	855	5.486E-09	***
pvalue_(Right)_ =	6.85E-26	T1&T2	–	T2H	860	7.862E-10	***	861	3.927E-10	***
W_(Right)_ =	0.76	T1&T2H	–	T2	814	8.554E-06	***	820	3.914E-06	***
		T1&T2H	–	T2H	855	5.486E-09	***	861	3.927E-10	***
		T2	–	T2H	710	0.068256	n.s.	712	0.06048	n.s.

**Whole_hippocampal_body**		T1	–	T1&T2	631	1	n.s.	661	0.864	n.s.
X^2^_(Left)_ =	45.68	T1	–	T1&T2H	834	4.968E-07	***	846	5.4E-08	***
pvalue_(Left)_ =	2.87E-09	T1	–	T2	496	1	n.s.	566	1	n.s.
W_(Left)_ =	0.28	T1	–	T2H	793	9.331E-05	***	838	2.514E-07	***
		T1&T2	–	T1&T2H	614	1	n.s.	594	1	n.s.
X^2^_(Right)_ =	73.31	T1&T2	–	T2	316	1	n.s.	365	1	n.s.
pvalue_(Right)_ =	4.54E-15	T1&T2	–	T2H	758	0.0022982	n.s.	846	5.4E-08	***
W_(Right)_ =	0.45	T1&T2H	–	T2	202	0.864	n.s.	290	1	n.s.
		T1&T2H	–	T2H	692	0.19656	n.s.	765	0.001283	n.s.
		T2	–	T2H	812	1.093E-05	***	831	7.992E-07	***

**Whole_hippocampus**		T1	–	T1&T2	731	0.0173232	n.s.	797	6.134E-05	***
X^2^_(Left)_ =	89.54	T1	–	T1&T2H	844	8.122E-08	***	856	3.927E-09	***
pvalue_(Left)_ =	1.65E-18	T1	–	T2	753	0.0034258	n.s.	814	8.554E-06	***
W_(Left)_ =	0.55	T1	–	T2H	860	7.862E-10	***	861	3.927E-10	***
		T1&T2	–	T1&T2H	517	1	n.s.	378	1	n.s.
X^2^_(Right)_ =	108.88	T1&T2	–	T2	679	0.397872	n.s.	743	0.007344	n.s.
pvalue_(Right)_ =	1.26E-22	T1&T2	–	T2H	849	2.752E-08	***	861	3.927E-10	***
W_(Right)_ =	0.66	T1&T2H	–	T2	519	1	n.s.	687	0.2592	n.s.
		T1&T2H	–	T2H	839	2.104E-07	***	857	2.752E-09	***
		T2	–	T2H	816	6.61E-06	***	816	6.61E-06	***

In addition to the 19 regions of interest, whole hippocampal head and body as well as whole hippocampal volume are presented. Significance level was set to *p* < 0.001 (***p-values presented were Bonferroni corrected for all pairwise tests). T2H was excluded for the hippocampal tail, as not the entire structure was covered due to the limited size of the field of view. T2: T2-weighted standard resolution; T2H: T2-weighted high resolution, GC-ML-DG: Granule cell and molecular layer of the dentate gyrus, HATA: Hippocampus-amygdala-transition-area; n.s., non significant.

**FIGURE 3:**
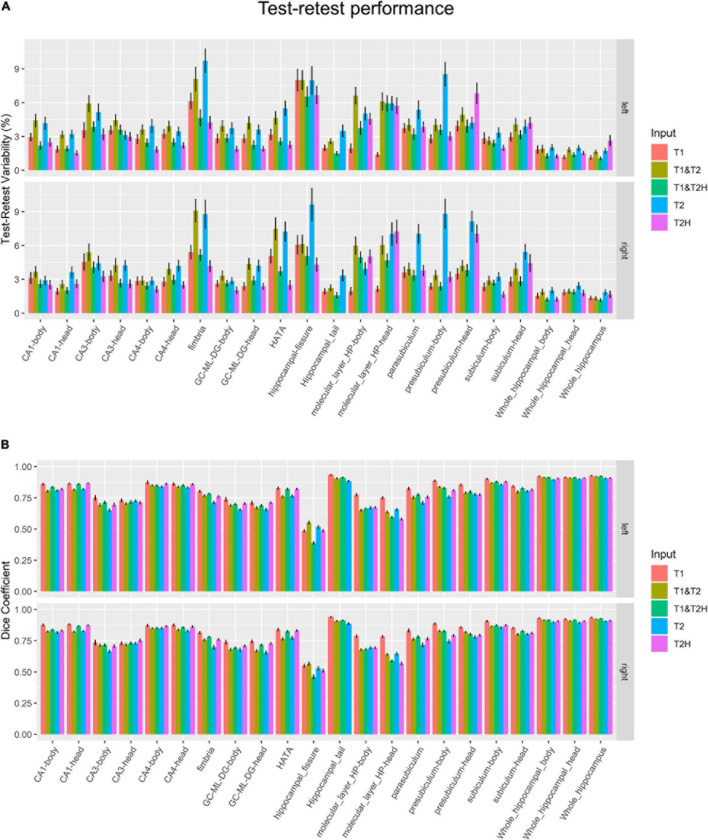
Longitudinal test-retest performance measurements. **(A)** Test-retest variability in percent and **(B)** dice coefficient metrics for each subfield and processing mode. T2H was excluded for the hippocampal tail, as not the entire structure was covered due to the limited size of the field of view. Error bars indicate standard error of the mean. T2, T2-weighted standard resolution; T2H, T2-weighted high resolution; GC-ML-DG, granule cell and molecular layer of the dentate gyrus; HATA, Hippocampus-amygdala-transition-area.

The test-retest metrics indicated best %TRV results (Figure 3A) across all subfields for T1 (3.24 ± 1.33) and T1 and T2H (3.30 ± 1.13), followed by T2H (3.47 ± 1.60). Higher variability was found for T1 and T2 (4.60 ± 1.61) and T2 alone (5.14 ± 2.01). However, these observed values differed drastically between the investigated ROIs and each area showed their own specific profile. For example, while T2H alone performed better or at least as good as T1 and T2H in several subfields, poor results were found in the presubiculum head (T2H: 6.94 ± 4.58, for comparison: T1 and T2H: 3.85 ± 2.57). On the other hand, T2 and the combination of T1 and T2 showed worst performance measures in almost all subfields. Especially weak %TRV results for T2 were found in the fimbria (9.24 ± 5.89), the presubiculum body (8.68 ± 6.33) and in the parasubiculum (6.20 ± 4.31).

These results mainly coincided with dice similarity coefficient (Figure 3B), where best metrics were found for T1 (0.81 ± 0.09) followed by T1 and T2H (0.77 ± 0.12). Slightly inferior, but almost identical results were observed for T2H (0.76 ± 0.10) and T1 and T2 (0.76 ± 0.09). As for the %TRV results, T2 performed not as good as the other approaches (0.74 ± 0.09). However, differences to other pipelines, except for T1, were not severe. Again, results varied strongly across the specific subfields. While in some regions almost no differences were observed between the processing modes, such as in CA4, T1 clearly showed better dice coefficients in contrast to all other approaches but also especially to the overall second best approach in the molecular layer body (T1: 0.78 ± 0.05; T1 and T2H: 0.67 ± 0.03), molecular layer head (T1: 0.77 ± 0.05; T1 and T2H: 0.59 ± 0.03), parasubiculum (T1: 0.83 ± 0.05; T1 and T2H: 0.78 ± 0.04) and in the presubiculum body (T1: 0.89 ± 0.03; T1 and T2H: 0.83 ± 0.03) and head (T1: 0.86 ± 0.03; T1 and T2H: 0.80 ± 0.04) for example. All results are presented with averaged left and right mean values of both hemispheres. In addition, to gain the high resolution for the T2H condition the FOV was economically chosen and for some participants the hippocampal tail was not entirely covered. Hence, this area was not included for the T2H condition in the summary statistics described above.

## Discussion

In this investigation, five different hippocampal subfield processing configurations were assessed and compared in a cross-sectional and longitudinal manner. Our results showed significant volume estimation differences between the used modes (T1, T2, T2H, T1 and T2, and T1 and T2H) in several subfields when compared cross-sectionally. Differences were most pronounced in the molecular layer (head), CA1 (head), hippocampal fissure, CA3 (head and body), fimbria and CA4 (head). In some of those areas, volume estimations between the processing types differed drastically, particularly in the head of the molecular layer with significant results between all pairwise comparisons, except for T1 vs. T1 and T2. Our results indicate a strong influence of the chosen pipeline on hippocampal subfield segmentation volume estimations.

The longitudinal analysis using %TRV and dice coefficient measurements revealed that T1 and multispectral analysis (T1 and T2H) showed better performance than T2H alone when all subfields are taken into consideration. However, the specific subfields had a substantial influence on the performance of segmentation results, regardless of the processing mode. For example, CA1, CA4, hippocampal tail (note that T2H was excluded from this region) and subiculum delivered excellent test-retest metrics for %TRV and dice coefficient measurements across the processing modes as observed in Whelan et al. (2016). Nevertheless, as observed in the cross-sectional investigation, subfield specific differences regarding the processing modes are highly apparent. The lowest test-retest performances were observed in the hippocampal fissure and the fimbria across all possible input variations, corroborating results from prior studies, where unispectral T1-weighted input at a standard resolution of around 1 mm^3^ had been used (Marizzoni et al., 2015; Whelan et al., 2016; Worker et al., 2018; Brown et al., 2020). In general, the volume estimations in these subfields must be interpreted with caution, as especially small hippocampal regions are harder to detect by the segmentation algorithm. It has been shown that larger hippocampal structures, such as the CA1, lead to more robust results (Marizzoni et al., 2015) also in comparison to manual delineations (Van Leemput et al., 2009). Our analyses suggest that even high-resolution T2 and the combination of T1 and T2H face difficulties in these smaller regions. Nevertheless, T2H exhibits better overall contrast properties to even detect subtle differences between the hippocampal structures, which cannot be accomplished with standard T1 resolution (Wisse et al., 2014). This was also corroborated by a recent study, indicating that high resolution T2 outperforms T1 in detecting atrophy in terms of effect sizes (Mueller et al., 2018). However, we could not detect better performance for high-resolution T2 in our reliability analysis when overall performance across all subfields was investigated. T2 and T1 and T2 showed the overall worst reliability measures, but especially in the fimbria, HATA, parasubiculum, and the presubiculum compared to the other options. In general, our results indicate no benefit in using either the standard resolution T2 sequence nor the combination of T1 and T2 compared to the default T1 processing stream.

Although the T1-weighted sequence with standard resolution of 1 mm^3^ delivered overall better test-retest metrics than T2H and T1 and T2H, several hippocampal substructures are only reliably detected using high resolution T2 or multispectral contrasts (T1 and T2H). Therefore, the gained segmentation results should be interpreted with caution, as results do not always reflect the underlying structures of the hippocampus (Wisse et al., 2020). In our analysis, an interesting observation was made for the head and the body of the molecular layer, where T1 showed best results for both test-retest metrics in comparison to all the other modes. A possible explanation why the test-retest results are fairly good in this region, is the fact that the algorithm relies heavily on prior information of the atlas when only the T1 sequence is used (Iglesias et al., 2015). Using the T1 standard resolution, the internal boundaries are not reliably detected and rely heavy on prior information of the atlas. This is especially true for the molecular layer, which cannot be detected reliable and relies on prior information (Iglesias et al., 2015; Giuliano et al., 2017). In addition, partial volume effects and signal variations have also be taken into consideration in the hippocampus, especially at such small substructures (Tohka, 2014; Worker et al., 2018). For the whole hippocampus, slightly better results were observed for T1 and T2H in comparison to T1 regarding %TRV.

FreeSurfer was used in this investigation, as it is freely available and widely used for brain segmentations including subfield parcellation of several subcortical structures. However, next to FreeSurfer, other hippocampal subfield segmentation tools exist while a recently published approach (LASHiS) seems to be a reasonable alternative. Especially at ultra-high fields, as it specifically supports longitudinal multispectral processing (Shaw et al., 2020). This is a drawback for FreeSurfer that longitudinal hippocampal processing is only possible using a T1-weighted image and not available for multispectral contrast inputs. This should be addressed in future releases of this software package as it was recently shown that the longitudinal approach outperformed cross-sectional hippocampal processing (Chiappiniello et al., 2020). In this investigation, authors also used a multispectral approach, however, focusing on the recon-all stream and not directly on the hippocampal subfield tool, as we did in our analysis. Furthermore, it is a vivid and ongoing debate how hippocampal subfield borders are defined and based on which criteria borders are delineated. No unified segmentation scheme is used by the scientific community. This is also problematic when several subfield tools are compared to each other or to postmortem measurements, as borders are defined according to different protocols. However, efforts are made by the Hippocampal Subfields Group (HSG) to unify the protocols and to develop a standardized method (Olsen et al., 2019). In addition, integrating cytoarchitecture, neuroreceptor information, and connectivity-based parcellations will deliver a more profound picture of this very homogenous brain structure (Plachti et al., 2019; Palomero-Gallagher et al., 2020).

If time is a limiting factor, acquiring only a T1 and running the parcellation with this sequence is a viable option, which might be even beneficial in certain subfields. However, our results indicate that one needs to be aware that the type of input images drastically changes the output. Regarding the reliability, T1 with standard resolution outperformed other sequences in distinct subfields, however, implicating the risk that results are biased, as mainly *a priori* information of the atlas is used (Iglesias et al., 2015).

Of note, given the small FOV of the high-resolution T2 sequence, in some of our subjects, the hippocampal tail was not entirely covered. Hence, we accounted for that fact and did not include T2H in the tail subfield. This is an issue one should be aware of as this may happen at those sequences with small FOVs to gain higher resolution. Here, no manual segmentation has been carried out in addition to the automatic assessment. Manual delineation is highly time consuming and especially in large datasets not an option. In addition, expertise of anatomy is needed and rater bias plays a role leading to problems of reproducibility across different centers (Wisse et al., 2016; Mueller et al., 2018).

Taken together, here we delivered a systematic comparison of available hippocampal processing input sets within the new FreeSurfer tool and assessed their performance using healthy young individuals. Future work may also investigate the performance in older cohorts or in patients with neurological conditions. Although T1 alone showed reliable results for the test-retest measurements, we advise to use high resolution T2 or multispectral information where T1 and high-resolution T2 is combined as it better reflects the underlying biological substrate by using high resolution and improved contrast properties.

## Conclusion

In this study, we measured a relatively large study cohort of 41 participants with three different MRI sequences (T1-, T2- and high-resolution T2-weighted) to assess the performance of five hippocampal segmentation modes within FreeSurfer. Our results revealed strong subfield volume estimation differences between the used pipelines, which has to be taken into account when segmentation results are compared between studies, where different approaches have been used. The greatest differences according to effect sizes were observed in the head of the molecular layer, CA1 head, hippocampal fissure, head and body of CA3 and fimbria. Our reliability analysis indicated overall good results for T1, T1 and T2H, and T2H. However, the usage of T1 at standard resolution relies heavily on prior information of the atlas and hardly reflects the underlying neurobiological complex structure of the hippocampus. Finally, and as expected, T2 or the use of multispectral T1 and T2 does not bring any beneficial effect and showed worst test-retest results. These findings are of particular importance when comparing results of previous studies using different segmentation schemes and once again call for detailed reports on data acquisition and processing, as well as a unified state-of-the-art approach.

## Data Availability Statement

The datasets presented in this article are not readily available because raw MRI data of participants used in this manuscript cannot to be shared due to ethical reasons. However, analyzed data sets are available. Requests to access the datasets should be directed to RL, rupert.lanzenberger@meduniwien.ac.at.

## Ethics Statement

The studies involving human participants were reviewed and approved by Ethical committee of the Medical University of Vienna. The patients/participants provided their written informed consent to participate in this study.

## Author Contributions

RS conducted the analyses, performed MR measurements, and wrote the manuscript. GMG, PH, JU, GG, and TV were responsible for the medical aspects of this study. FH, MR, BS-D, and MK were involved in data analyses and/or conducted the MR measurements. RL supervised the entire procedures and served as principal investigator. All authors read and commented on the manuscript and gave approval for publication in its current form.

## Conflict of Interest

With no relevance to this work, RL received travel grants and/or conference speaker honoraria within the last 3 years from Bruker BioSpin MR, Heel, and support from Siemens Healthcare regarding clinical research using PET/MR. RL is a shareholder of the start-up company BM Health GmbH since 2019. The remaining authors declare that the research was conducted in the absence of any commercial or financial relationships that could be construed as a potential conflict of interest.

## Publisher’s Note

All claims expressed in this article are solely those of the authors and do not necessarily represent those of their affiliated organizations, or those of the publisher, the editors and the reviewers. Any product that may be evaluated in this article, or claim that may be made by its manufacturer, is not guaranteed or endorsed by the publisher.

## Footnotes

1
http://surfer.nmr.mgh.harvard.edu
